# Craniometric Analysis of the Hindbrain and Craniocervical Junction of Chihuahua, Affenpinscher and Cavalier King Charles Spaniel Dogs With and Without Syringomyelia Secondary to Chiari-Like Malformation

**DOI:** 10.1371/journal.pone.0169898

**Published:** 2017-01-25

**Authors:** Susan P. Knowler, Anna-Mariam Kiviranta, Angus K. McFadyen, Tarja S. Jokinen, Roberto M. La Ragione, Clare Rusbridge

**Affiliations:** 1 School of Veterinary Medicine, Faculty of Health & Medical Sciences, University of Surrey, Guildford, Surrey, United Kingdom; 2 Department of Equine and Small Animal Medicine, 00014 University of Helsinki, Finland; 3 akm-stats, Glasgow, Scotland, United Kingdom; 4 Fitzpatrick Referrals, Godalming, Surrey, United Kingdom; Faculty of Animal Sciences and Food Engineering, University of São Paulo, BRAZIL

## Abstract

**Objectives:**

To characterize and compare the phenotypic variables of the hindbrain and craniocervical junction associated with syringomyelia (SM) in the Chihuahua, Affenpinscher and Cavalier King Charles Spaniel (CKCS).

**Method:**

Analysis of 273 T1-weighted mid-sagittal DICOM sequences of the hindbrain and craniocervical junction from 99 Chihuahuas, 42 Affenpinschers and 132 CKCSs. The study compared 22 morphometric features (11 lines, eight angles and three ratios) of dogs with and without SM using refined techniques based on previous studies of the Griffon Bruxellois (GB) using Discriminant Function Analysis and ANOVA with *post-hoc* corrections.

**Results:**

The analysis identified 14/22 significant traits for SM in the three dog breeds, five of which were identical to those reported for the GB and suggest inclusion of a common aetiology. One ratio, caudal fossa height to the length of the skull base extended to an imaginary point of alignment between the atlas and supraoccipital bones, was common to all three breeds (p values 0.029 to <0.001). Associated with SM were a reduced occipital crest and two acute changes in angulation i) ‘sphenoid flexure’ at the spheno-occipital synchondrosis ii) ‘cervical flexure’ at the foramen magnum allied with medulla oblongata elevation. Comparing dogs with and without SM, each breed had a unique trait: Chihuahua had a smaller angle between the dens, atlas and basioccipital bone (p value < 0.001); Affenpinschers had a smaller distance from atlas to dens (p value 0.009); CKCS had a shorter distance between the spheno-occipital synchondrosis and atlas (p value 0.007).

**Conclusion:**

The selected morphometries successfully characterised conformational changes in the brain and craniocervical junction that might form the basis of a diagnostic tool for all breeds. The severity of SM involved a spectrum of abnormalities, incurred by changes in both angulation and size that could alter neural parenchyma compliance and/or impede cerebrospinal fluid channels.

## Introduction

Syringomyelia (SM) secondary to Chiari-like malformation (CM) is diagnosed increasingly in toy breed dogs [1]. Since both of these conditions can be accompanied by pain and /or neurological deficits [2–4], there are associated welfare concerns and a desire to reduce the disease prevalence in predisposed breeds [5]. CM, sometimes referred to as caudal occipital malformation syndrome (COMS), is a complex developmental abnormality of the skull and craniocervical junction. It is usually defined as a mismatch between size of the brain and the caudal fossa leading to cerebellar and brainstem herniation [6–8]. Recently other research studies have reported that CM is characterized by the shortening of the skull base, reduced caudal cranial fossa volume and increased proximity of the cranial cervical vertebrae to the skull [9, 10]. The mismatch of the skull and brain, like its human equivalent Chiari type I and O malformation (CM1 and CM0) [11], disrupts the cerebrospinal fluid (CSF) flow dynamics which may be progressive over time [12–14] with resultant CSF-like fluid cavitation in the spinal cord known as SM [15, 16].

The Cavalier King Charles Spaniel (CKCS) has a high prevalence for CM/SM which has been well documented over the last decade and contributed to a considerable knowledge base for these conditions [17]. CM is ubiquitous in the CKCS breed and it is likely that additional risk factors account for a predisposition to SM [18]. However, in a recent study investigating the prevalence of CM in clinically unaffected dogs, it was concluded that the high prevalence of cerebellar indentation and impaction in toy breeds may be “normal” anatomical variations [19]. The inheritance of CM/SM is complex [20, 21] and it has been shown to have a multifactorial relationship between CM and SM in the CKCS [22, 23] and the Griffon Bruxellois (GB)[9, 24].

Two particular risk factors associated with CM/SM are miniaturization and brachycephaly [25–27]. The Chihuahua is the smallest known dog breed for which SM has been reported. Both British and American breed standards state that a Chihuahua must not weigh more than 2.7 kg (6 lb). An investigation of this miniature breed may therefore elucidate the pathogenesis of CM/SM. The Affenpinscher is a brachycephalic dog, often referred to as the “monkey dog”. It is quite similar in appearance to the GB but typically larger and with a less stocky build. Although the Affenpinscher wirehaired head has rostrocaudal shortening similar to the GB, its muzzle is not as flattened [28] and provides an interesting brachycephalic variation for CM/SM investigation.

### Study objectives

This study compares Magnetic Resonance images (MRI) taken in the mid-sagittal plane obtained from three breeds, the CKCS, Affenpinscher and Chihuahua, using refined morphometric techniques that were originally developed to investigate the genetic basis of CM in the Griffon Bruxellois (GB). The study aims were:

1. Characterize the phenotypic variables of the caudal fossa and craniocervical junction associated with SM (i.e. characterize the phenotypic variables of canine CM).
2. Elucidate any conformation similarities between the breeds that might suggest a common aetiology and assist in diagnosis.
3. Identify any protective conformation traits that might contribute to generating estimated breeding values in order to reduce the risk of SM through selective breeding.

### Ethics statement

This retrospective study analysed Digital Imaging and Communications in Medicine (DICOM) data obtained from dogs that underwent MRI either for diagnostic purposes for assessment of CM/SM status prior to breeding or for diagnostic investigation of neurological signs and / or pain and was approved by the local ethics committee at the University of Surrey (reference NASAP-2015-001-SVM).

## Materials and Methods

### MRI DICOM data

This investigation comprised a total of 273 T1-weighted (T1w) DICOM sequences in the mid-sagittal plane of the hindbrain and craniocervical junction of which 132 were CKCS, 42 Affenpinschers and 99 Chihuahuas. These were acquired from dogs undergoing either diagnostic investigation that included T1w imaging of the hindbrain and craniocervical junction or screening for CM/SM prior to breeding. The imaging data was obtained from databases at the Stone Lion Veterinary Hospital (SLVH) and Fitzpatrick Referrals Ltd (FR) or sent to CR in support of the research into CMSM. The Chihuahua cohort included 53 DICOMS obtained from the Veterinary Teaching Hospital of University of Helsinki (VTHUH) that had participated in a low cost screening examination for CMSM with voluntary participation of owners which was part of another study (paper under writing process). SLVH and VTHUH used a 0.2T MR scanner (Esaote S.p.A Genova, Italy) where the dogs were positioned in sternal recumbency. FR used a 1.5T scanner (Siemens Symphony Mastro Class, Enlargen, Germany) and positioned the dogs in dorsal recumbency. Any images of the dog’s head in the flexed position or misaligned in the coil were excluded from the study. T1w images were selected, in keeping with the previous GB study, because they provided the optimum resolution for bone density [29].

### Study cohort

Each breed was grouped according to its SM status based on the British Veterinary Association (BVA) /Kennel Club (KC) CM/SM Health screening scheme [6]. CKCS and Affenpinschers that were not graded by the BVA were evaluated by CR (a BVA/KC CMSM Health Scheme scrutineer). A-MK examined and imaged 53/99 Chihuahuas and their SM status was evaluated jointly by CR and TSJ who were blinded to their clinical status.

SM is defined as a CSF like -fluid filled cavity that includes, or is distinct from, the central canal of the spinal cord and graded according to its maximum internal diameter in a transverse plane. SM0 has no syrinx or central canal dilation. SM1 has a central canal dilatation with an internal diameter less than 2mm. SM2 has a fluid filled cavity ≥ 2mm or pre-syrinx. SM is usually a progressive condition [12, 30] and although the SM status for a late onset condition could not be confirmed in dogs less than 5 years, young dogs without SM could be matched with dogs that did have SM of a similar age. Neither the clinical status of the dogs nor the BVA/KC evaluations of CM (based on the shape of the cerebellum) were considered variables in this particular investigation. Table 1 provides details of the group composition.

**Table 1:** Study cohort. Chi = Chihuahua; Affens = Affenpinscher.

		TOTAL BREED GROUP			
		Chi	Affens	CKCS	Total number dogs
**gender**	**male**	51	16	48	**115**
	**female**	48	26	84	**158**
**age group**	**<3yrs**	32	11	36	**79**
	**3–4.9yrs**	34	15	32	**81**
	**≥5yrs**	33	16	64	**113**
**SM grade**	**0**	34	28	45	**107**
	**1**	23	6	18	**47**
	**2**	42	8	69	**119**
**Group**	**Total**	**99**	**42**	132	**273**

### Morphometric measurements

T1w mid-sagittal brain and cranial cervical spinal cord images were anonymised and all measurements taken by SPK, initially blinded to clinical status, using a DICOM reading software package Mimics® 14.12 Materialise (15 3001 Leuven Belgium).

**Fig 1:**
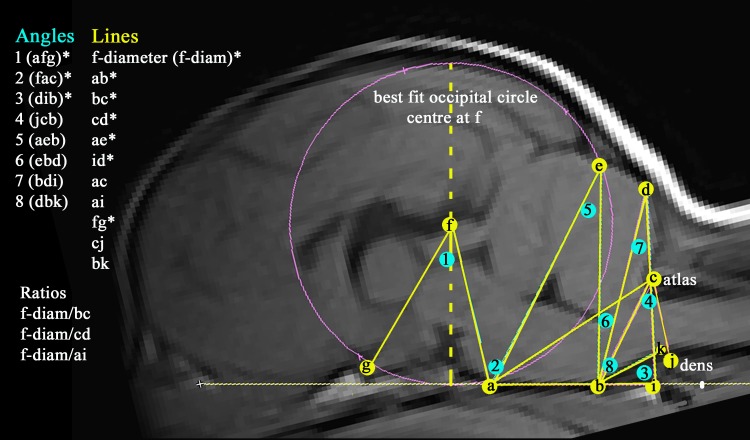
T1w sagittal MRI of a five year old Chihuahua without SM with a framework of 19 measurements (11 lines and 8 angles) with three ratios used to ‘map’ the hindbrain and craniocervical junction. Key: (a) dorsum of spheno-occipital synchondrosis. (b) basion of basioccipital bone. (c) rostral edge of the dorsal lamina of the atlas. (d) junction between the supraoccipital bone and the occipital crest. (e) most dorsal point of intersection of the cerebellum with the occipital lobe circle. (f) centre of’ best fit’ occipital lobe circle placed on the cranial baseline (abi) and extending to encompass the occipital lobes. The centre of the circle is f and its diameter (f-diam) indicates the maximun height of the caudal calvaria dorsal to the spheno-occipital synchondrosis. (g) point at which the optic nerve deviates into the optic canal. (i) intersection point of the extended cranial baseline (ab) caudally with extended line dc ventrally to form angle 3. This indicates the relative positions of the supra and basioccipital bones to the atlas. (j) most rostral aspect of the dens of the axis bone. (k) extended line from point b along the best fit line of the ventral medulla oblongata to where it changes angle to the spinal cord (degree of medullary kinking). 8 angles measured are (1) afg, (2) fac, (3) dib, (4) jcb (5) aeb (6) ebd (7) bdi (8) dbk. * trait used in previous GB study [9].

Fig 1 illustrates the 19 measurements taken (11 lines and 8 angles) and used to construct a ‘grid framework’ to explore the caudal fossa and craniocervical junction and record differences in the juxtaposition of hindbrain, spinal cord and skull in dogs with and without SM.

Measurements used in the original GB investigation [9] (marked *) were augmented to include:

1. The position of the odontoid process (dens) relative to the atlas since this was hypothesised to impact on the degree of craniocervical stenosis, angling of the medulla and/or obstruction of CSF channels.
2. Additional triangulation of angles arising from the basicranium to landmarks in the caudal fossa to reflect any overcrowding of the cerebellum and medulla oblongata.

### Statistical analysis

IBM SPSS for Windows® version 22 was used to calculate measurement reliability (Intraclass Correlation Coefficient (ICC) model). P-values were considered significant ≤0.05; Discriminant Analysis (DA) is a statistical technique used to determine the most important phenotypic trait variables which distinguish each group. In such an analysis the selected traits are evaluated by using cross-validation to avoid data bias and to confirm the prediction model. DA technique takes account of any correlations between variables and how reliable these are for predicting the group to which each dog had been assigned.

Analysis of Variance (ANOVA) with a *Post Hoc* Tukey correction for multiple testing which allows for the disparity in sub group sizes within each individual breed. Box plots with superimposed means plots were generated of significant traits between the breeds and the SM status and visualize the comparisons.

### Reliability analysis

In order to ensure consistency, only SPK undertook data measurement, blinded to the SM status graded by the evaluators. Intra-observer reliability measurements of 2 lines, 2 angles and 1 circle from ten dogs were obtained by SPK and these measurements repeated one year later.

## Results

Intra-observer reliability was high with all ICC values in excess of 0.75 with an average value 0.86.

### Statistical analysis

#### Total cohort

**Fig 2:**
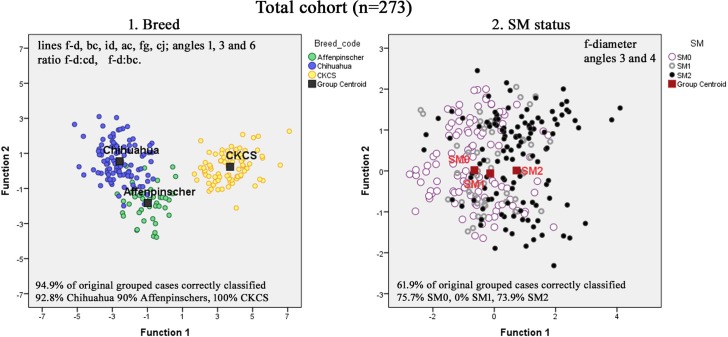
Scatterplots for canonical Discriminant Functions Analysis for total group cohort. Discriminant analysis is used to determine the minimum number of dimensions needed to describe differences between the group for (1) Breed and (2) SM. These significant variables identified are allocated a weight within each discriminant function (S2 Table) and the two functions plotted against each other to illustrate group seperation. The 11 traits can distinguish with 100% accuracy for CKCS, 92.8% for Chihuahuas and 90% for Affenpinschers. Separation in SM status using 3 traits yields 75.7% accuracy for SMO, 73.9% for SM2 but 0% for SM1 predictive success for each group.

Since the three breeds differ markedly in size and head shape, an initial DA investigation of the 19 variables and three ratios was performed to determine those that best discriminated between the three breeds. It revealed that a minimum of six lines, three angles and two ratios could be used to correctly classify an average of 94.4% of the dogs when cross-validated (Fig 2). The f-diameter of the ‘best fit’ occipital circle (f-diam), Angles 3 and 4 were identified as the most significant variables that distinguished between the groups when the breeds were combined and SM status substituted as the independent variable in the multivariate analysis. However there was only a 61.9% successful prediction for correct SM classification (Fig 2). Scatterplots for both canonical DA are provided in Fig 2. S1 Table lists the most significant traits used in the construction of the plots.

#### Individual breeds

**Fig 3:**
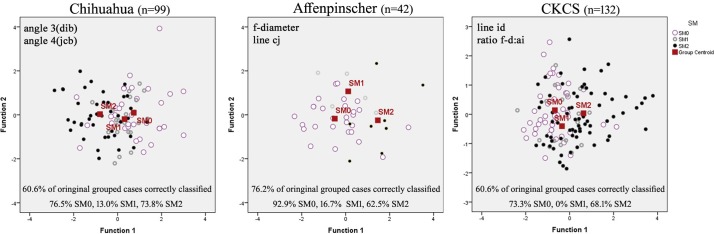
Scatterplots for canonical Discriminant Analysis for three breeds, Chihuahua, Affenpinscher and CKCS. **Chihuahua**: Left to right, there is less risk of SM left to right as angle 3 decreases and angle 4 increases (atlas further caudal from the supraoccipital and basioccipital bones) **Affenpinscher:** Left to right, increased risk of SM with decreasing line cj and increasing f-diam) **CKCS**: Left to right, increasing risk of SM with increasing line id and ratio f-diam: ai (i.e. dogs that are more brachycephalic).

Scatterplots, generated when DA was applied to each breed, identified different significant traits that were most important for each cohort (Fig 3). In the Chihuahua this was Angle 3 (dib) and Angle 4 (jcb) both which relate to the craniocervical junction and the proximity and alignment of the atlas and dens with the basioccipital bone (skull base). Affenpinschers appear similar to the GB in that the most useful trait was f-diam which is increased with SM. However the Affenpinschers are unique in that it is the distance of the dens to the atlas that best separates the subgroups with 92% correctly placed for SM0. In the CKCS scatterplot, line id (distance between the occipital crest and the level of the cranial baseline abi) is plotted against ratio f-diam/bc (the height of the caudal cranial fossa / distance of the atlas from the basioccipital across the foramen magnum). **S2 Table** gives the function coefficients used in the analysis.

SM is a late onset condition and the status of the SM1 dogs (central canal dilatation less than 2mm) cannot be confirmed less than 5 years old. Of the total SM0 dogs for each breed, 19% (8/42) Affenpinschers, 12% (12/99) Chihuahua and 3% (4/132) CKCS were under three years old and 21% (9/42) Affenpinschers, 8% (8/99) Chihuahua and 7% (9/132) CKCS were three years to less than five years old. The phenotypes of these relatively low numbers of SM0 dogs with unknown status were useful to compare those with SM1 and SM2 dogs of a similar age. The ambiguity of subgroup SM1 is demonstrated in Fig 3 with low percentage predictive scores of the original groups classified as correct (13% Chihuahuas, 16% Affenpinschers and 0% CKCS).

The total 14 significant variables for SM identified by ANOVA analysis after Tukey correction are provided in Table 2. These included the 5 traits * found in the previous GB analysis supporting the idea of a common aetiology.

**Table 2:** Significant variables for SM status identified in ANOVA after Tukey correction. * Traits identified as significant in previous GB study. *L =* angle. Significant p values ≤ 0.05 for SM affectedness are highlighted in bold.

		Chihuahua		Affenpinscher		CKCS	
no	variable	F	p-value	F	p-value	F	p-value
1	**bi**	7.294	**0.001**	1.692	0.197	11.516	**<0.001**
2	**ai**	6.455	**0.002**	1.860	0.169	10.982	**<0.001**
3	**bk**	8.966	**<0.001**	0.582	0.563	3.802	**0.025**
4	***L*7 (bdi)**	8.819	**<0.001**	2.126	0.133	13.217	**<0.001**
5	*L*3(dib)*	9.406	**<0.001**	2.847	0.070	14.548	**<0.001**
6	**f-diam:bc**	8.957	**<0.001**	2.307	0.113	9.463	**<0.001**
7	**cj**	0.711	0.494	5.390	**0.009**	1.511	0.225
8	bc*	4.306	**0.016**	3.566	**0.038**	2.319	0.102
9	ae*	1.802	0.170	3.248	**0.050**	5.408	**0.006**
10	f-diam*	2.106	0.127	6.991	**0.003**	8.210	**<0.001**
11	***L*4(jcb)**	16.770	**<0.001**	0.394	0.677	2.711	0.070
12	fg*	4.443	**0.014**	0.830	0.444	0.650	0.525
13	**ac**	1.696	0.189	0.497	0.612	5.220	**0.007**
14	**f-diam:ai**	8.446	**<0.001**	3.884	**0.029**	15.329	**<0.001**

**Fig 4:**
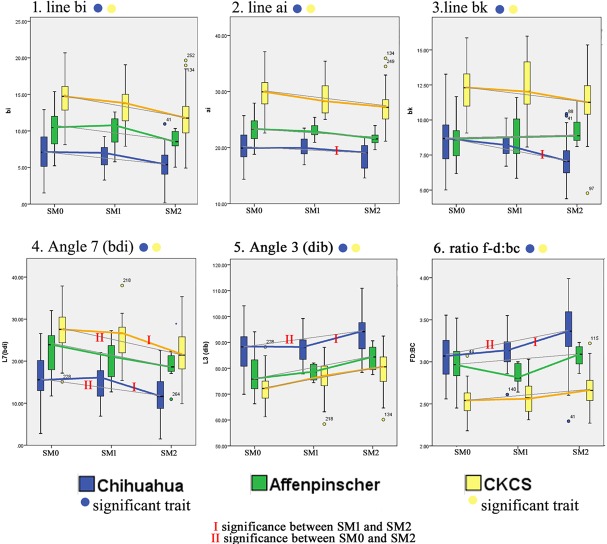
Descriptive boxplots of variables associated with SM status significant for both Chihuahua and CKCS cohorts. The mean values for SM0, SM1 and SM2 have been linked with a colour coded line for each breed. In addition, a thin grey line has been drawn between mean values SM0 directly to SM2. Unless indicated by red bar/s, the significance is between SM0 and SM2.

**Fig 5:**
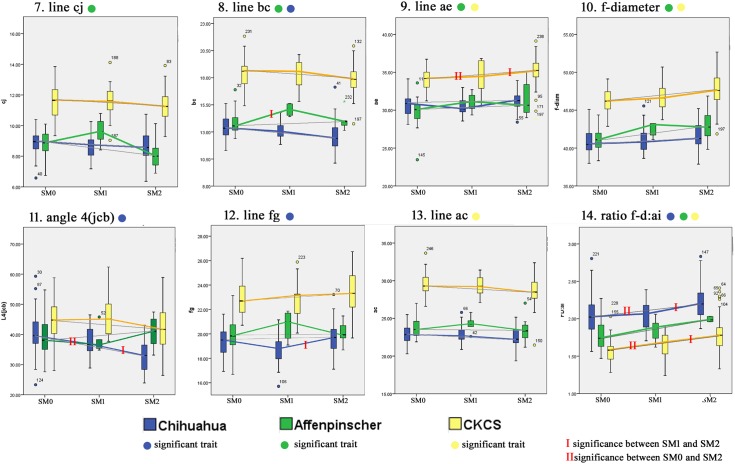
Descriptive boxplots of significant variables associated with SM status in the Affenpinscher, Chihuahua and CKCS. The mean values for SM0, SM1 and SM2 have been linked with a colour coded line for each breed. In addition, a thin grey line has been drawn between mean values SM0 directly to SM2. Unless indicated by red bar/s, the significance is between SM0 and SM2.

S3 Table lists the complete data used in the study. Descriptive boxplots highlighting the significant morphological differences and similarities that were identified in the univariate analysis have been grouped into two figures to ease reading (Fig 4 and Fig 5). Colour coded circles indicate which trait is significant for the breed. Since the breeds are plotted on the same axis for each trait, the differences in size between breeds is apparent: the largest CKCS (yellow), the smallest Chihuahua (blue) and the Affenpinscher (green) in between. The mean values for SM0, SM1 and SM2 have been linked with a colour coded line for each breed. However since the statistical analysis identified significance between SM0 and SM2, a thin grey line has been drawn between their mean values in order to accentuate differences between the two or disparity with SM1. A red bar has been added to the coloured line if the significance was between SM0 and SM1 (only example line bc) or SM1 and SM2 (nine examples). A double red bar has been added if there was an additional significance between SM0 and SM2 (eight examples). Fig 4 illustrates six traits that are significant in both the Chihuahua and the CKCS breeds and Fig 5 three traits shared by the remaining traits (Table 2) shared by the Affenpinschers or those unique to each breed.

#### Morphometric analysis

**Fig 6:**
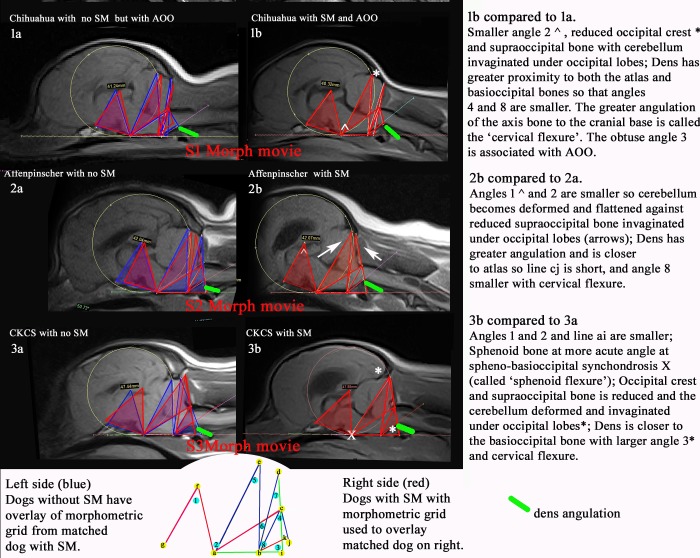
Four pairs of TW1 mid- sagittal MRI of exemplar Chihuahua, Affenpinscher and CKCS with and without SM and their morphometric overlays. The pairs of dogs have been matched as far as possible in age and size of the occipital circle (f-diam) which is typically larger in Affenpinschers and CKCS dogs with SM. AOO = atlanto-occipital overlap. Note the reduced occipital crest in all 3 dogs with SM (a) and marked ventriculomegally in SM dogs 2b and 3b. The images in each pair have been morphed with each other to provide a dynamic illustration (S1–S3 Morph Movies).

Fig 6. compares the morphometric grids for three exemplar breed pairs; Chihuahua, Affenpinscher and CKCS. Each pair of sagittal MR images is matched as far as possible for age and the diameter of its occipital circle (f-diam) but opposing SM status. SM2 (affected) red coloured grid is superimposed over that of the SM0 image (unaffected blue grid), highlighting the discrepancies between the paired dogs.

All the SM affected dogs (b) have a smaller line ac, cj and angles 2 and 4 but they vary in degrees and the proximity and angulation of the atlas and dens. For example, dogs 1b and 3b have the same length line bc as their counterparts but a smaller angle 7 and medullary kinking associated with a smaller angle 8 (dbk). Despite the breeds’ differences in morphometric proportions, Fig 6 illustrates the cumulative similarities of the shared deformities associated with SM.

### Limitations of the study

Although this retrospective study included MR images from different sources the landmarks selected were those most easily identified irrespective of machine resolution and had been verified in previous studies. The effect of different positioning in the MR coil with respect to sternal or dorsal recumbency was not considered an issue because the head was fully extended in both positions. The poorer signal to noise ratio of low-field MRI inevitably makes interpretation of SM1 challenging but variability was limited by having the same interpreter for all the evaluations (CR) and more than one when possible.

The number of Affenpinschers affected with SM and number of young dogs of unconfirmed SM status compared to the other two subgroups was a limitation in the study. It was considered important to include all dogs possible and dogs without SM could be age matched for age with dogs with SM. Affenpinschers are not as popular as CKCS and Chihuahua but the low numbers of reported symptomatic dogs may also be due to differences in head conformation in the breed that reduce the risk of SM.

Ventriculomegaly has been shown to be associated with SM [7] and although obvious differences in ventricular size were noted (Fig 6) these were not investigated in this particular study but part of an alternative ongoing investigation.

## Discussion

This study aimed to distinguish the phenotypic variables of the caudal skull, hindbrain and craniocervical junction in three different breeds and how these predispose the formation of one or more syringes in the spinal cord. Individual predisposing features, such as medulla oblongata elevation (kinking) [22, 31] and atlanto-occipital overlapping [32–34], have previously been evaluated in other studies using different parameters. They show to be closely associated with SM but are not an evitable consequence i.e. having this anatomical feature is not always associated with SM and vice versa. Equally, dens abnormalities have been reported [35] but a consistent association with SM remains unclear [10]. This study differs in that it quantifies the combined influence of all predisposing features including the reduced caudal fossa size and attempts to identify protective elements. The 19 selected morphological measurements were successful in providing a comprehensive means of analysing the proportions and juxtapositions of the hindbrain and craniocervical junction in the three breeds.

Seven significant traits identified in previous GB studies namely f-diam, line bc, ae, fg and angles 1 (in that study angle 5) 2 and 3 were also identified in this investigation and supports the basis of a shared aetiology of CM/SM [9, 36, 37]. Although there were differences in breed size (Fig 2) and absolute values (Fig 4 and Fig 5), the trends for SM0 to SM2 status were broadly similar in the three breeds.

The relationship between the traits is demonstrated by the S1–S3 Morph Movies. These dynamic illustrations reveal two flexures associated with SM that are not appreciated in the static DICOM images. The morph movies help illustrate how seemingly minor changes in skull and vertebral conformation can be additive towards distorting the neural parenchyma and thereby compromising the CSF flow dynamics both within the brain and the spinal cord. Furthermore, despite the dissimilar proportions in the morphometries of the three breeds, the underlying forces that are revealed in the movies appear similar but in different degrees.

### Breed characteristics for SM

#### Chihuahuas (S1 Morph Movie)

Angle 3(dib) and Angle 4 (jcb) were identified as the most discriminating factors in this breed (Fig 3). An increase in Angle 3 is associated with flattening and deformation of the cerebellum whereas Angle 4 flexure represents overcrowding at the craniocervical junction and neural parenchyma (Fig 6). The significant traits (p<0.001) line bk, angle 7 and ratio f-diam: bc also relate to the craniocervical junction. Small differences in volume reduction may have a greater impact in compromising the CSF flow into the subarachnoid space of the miniaturised Chihuahua compared to larger breeds with more leeway. An increase in cranial height (f-diam) was not a significant factor for SM in this breed. However the reduction of angles 4 and 7 and the considerable reduction in the occipital crest resulted in SM dogs having a more rounded and rostrocaudal short skull. Insufficiency of the caudal skull has been previously described as a feature of SM dogs in the GB [24, 36] and CKCS [27].

#### Affenpinschers (S2 Morph Movie)

This study showed that syringomyelia in the Affenpinscher is associated with increased proximity of the dens to the atlas i.e. reduced line cj and this feature was unique to the breed (p = 0.009). The steeper angulation of the dens was associated with flexure of the craniocervical neural parenchyma i.e. the medulla oblongata and spinal cord (Figs 6 and 2B). This can be compared to basilar invagination (BI) in humans which a common craniocervical junction malformation associated with CMI [38]. Classical BI in humans is defined as invagination of the odontoid process into the foramen magnum with ventral brainstem compression i.e. a more severe malformation. However angular craniometric studies have distinguished subgroups of BI in humans. Type II has invagination of the dens towards the skull but not inside the foramen magnum but has a more acute angulation between the floor of the caudal fossa and the dorsal dens (clivus canal angle; in humans the clivus is the posterior skull base, i.e. posterior sphenoid and basioccipital bone, and should be in line with the dens) and greater cervical lordosis (angle between dorsal dens and dorsal surface of c7 vertebral body) [39]. It is Type II BI that seems comparable to risk factors for SM affectedness in the Affenpinscher.

Noteworthy is the relationship of SM1 with both SM0 and SM2 that appears incongruous in the Affenpinscher compared to the two other breeds in Fig 5. It is possible that, in this breed, SM1 is not always an intermediate between SM0 and SM2 but associated with its own combination of conformation risk factors and SM1 does not necessarily progress to SM2 over time.

#### CKCS (S3 Morph Movie)

Ten of the fourteen significant variables were found in the CKCS with one, line ac, unique to the breed. Line ac indicates the proximity of the spheno-occipital synchondrosis to the atlas bone. This study confirms the findings of others that the CKCS with SM have a reduced caudal fossa size [17, 32, 40, 41] a presumed consequence of early closure of the spheno-occipital and possibly other cranial sutures[42]. Compared to other breeds including the GB [43], the CKCS has considerably greater incidence of cerebellar deformation by the supra-occipital bone and vermis herniation [19, 41, 44]. These findings and the coexistence of occipital dysplasia and hypoplasia [23] suggest that the CKCS may have additional predisposing risk factors for SM compared to the other breeds.

Figs 5, 3A and 3B illustrates the conformation differences in association with SM. Typically a CKCS with SM has an increased f-diam compared to those without SM (p value <0.001) but in this chosen example both dogs 3a and 3b have a similar f-diam ~47mm, thereby negating this variable. SM affected dog 3b has a reduced occipital crest and flattened supraoccipital bone. The atlas is more rostral and the dorsal atlanto-occipital membrane is vertically orientated. The cerebellum is deformed and flattened by the supraoccipital bone. There is only a small herniation of the cerebellar vermis into the spinal canal. Note the acute angle the sphenoid bone makes with the basioccipital bone of the SM affected dog 3b at the spheno-occipital synchondrosis. The superimposed red triangle of this dog on the blue triangle of unaffected dog 3a appears rotated caudally at point X. This elevated angulation of the sphenoid bone is called the ‘sphenoid flexure’ in this study. Additionally in this exemplar, the dens of dog 3b is both closer to the basioccipital bone and has greater angulation. This is comparable to an increased clivo-axial angle in humans and is called the ‘cervical flexure’ in this study. In humans ventral brainstem compression is associated with considerable pain and normalization of the clivo-axial angle with surgical intervention is linked with clinical improvement [45].

#### Pathophysiological basis of Chiari-like malformation and associated craniocervical junction abnormalities

**Fig 7:**
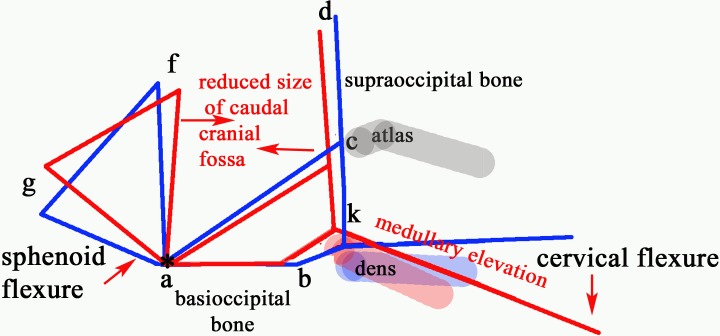
Schematic framework of selected CM traits (red lines) and ‘normal’ traits (blue lines) to illustrate underlying unifying pathophysiological processes. Key: (a) dorsum of spheno-occipital synchondrosis. (b) basion of basioccipital bone. (c) rostral edge of the dorsal lamina of the atlas. (d) junction between the supraoccipital bone and the occipital crest. (f) centre of “best fit” occipital lobe circle placed on the cranial baseline (abi) and extended to encompass the occipital lobes. The centre of the circle is f and indicates the height of the caudal cranial fossa. (g) point at which the optic nerve deviates into the optic canal. (k) extended line from point b along the best fit line of the ventral medulla oblongata to where it changes angle to the spinal cord (degree of medullary elevation/ kinking). The red (SM affected) lines are superimposed on those for representing normal (blue) and aligned along the skull base at fixed point ‘a’ dorsum of spheno-occipital synchondrosis (star). The basisphenoid, possibly the presphenoid, is flexed dorso-caudally (‘sphenoid flexure’) from the point of the synchondrosis (*) and the cranial cervical vertebrae especially the odontoid process that becomes angled ventrally and closer to the basioccipital bone with medullary elevation (‘cervical flexure’). Dogs may have one or more of the features which predispose to SM.

The genetic and endocrinal basis of craniosynostosis is pivotal in skull remodelling [46] and a correlation between patterns of closure and skull shape in the domestic dog has been demonstrated [47]. Central to CM is the reduced size of the caudal fossa and space for the craniocervical junction and cisterna magnum but the manner in which this abnormality is attained is variable. Fig 7 provides a diagrammatic summary of the key morphological traits identified in this study when comparing dogs with and without SM and illustrates the relationships between the variables

#### Reduced occipital crest and supraoccipital bone

An interesting observation in this study was the reduction of the occipital crest in SM dogs in all three breeds (Fig 6). Point ‘d’ in the framework grid is the junction of the occipital crest and the supraoccipital bone and its placement is an intrinsic part of angle 3, 6, and 7. Underdeveloped occipital bones have long been considered to play an influential role in CM and developing SM [48]. Unlike the other occipital bones, the supraoccipital bone ossifies by both intramembranous and endochondral means. This bone is derived from cephalic paraxial mesoderm, and possibly neural crest [49, 50], whereas the remaining occipital bones are somatic mesoderm origin [51]. A histopathological study of the neonatal supraoccipital bone in the CKCS showed that foetal tissue bone was poorer quality with significantly reduced number of osteoblasts and chrondrocytes with increased osteoclasts and apotosis compared to controls. In contrast, the adult CKCS supraoccipital bone had poor cellularity compared to controls. Furthermore the adult supraoccipital bone showed histological signs of active remodelling and it was hypothesised that this could alter the capacity to accommodate the mechanical pressure from the growing brain [52]. The four occipital bones that surround the foramen magnum and form part of the skull base, together with the sphenoid and the petrous temporal bones, are cartilaginous endochondral bone and mesodermal in origin (the chondrocranium) [53] unlike the bones of the skull vault are membranous and neural crest in origin (the dermatocranium) [54].

#### Changes in angulations which impact on the caudal cranial fossa

Another interesting finding in this investigation is the changes in angulations associated with SM and reduction of caudal cranial fossa volume; 1) ‘cervical flexure’ associated with changes in dens angulation and medullary elevation (kinking) at the craniocervical junction described in the Affenpinschers, 2) ‘sphenoid flexure’ at the spheno-occipital synchondrosis described in the CKCS. Change in craniocervical morphology as a consequence of a shortened and flattened clivus (i.e. sphenoid and basioccipital complex) is well described in humans and explained by different timings of sclerotome development. The fusion of the basioccipital and occipital sclerotomes occurs before the cervical sclerotomes and a lordotic skull base angle “forces” retroflexion of the cranial cervical segments and results in a dens that points up and back into the neural parenchyma [55]. However whether a similar situation occurs in dogs is yet to be established.

In rats, when the spheno-occipital synchondrosis was surgically removed it drastically changed the pattern of growth of the skull but tampering with the sutures of the vault did not. Compared to controls, the exorcized experimental rats had observable differences in the angulation of the skull base, an increased curvature of the cranial roof and a marked forward displacement of the occipital condyles. Other changes included a ventral and forward rotation of the plane of the foramen magnum [56]. It is hypothesised that the changes in angulation at the spheno-occipital and the spheno-presphenoid synchondroses (“sphenoid flexure”) occur during development. Furthermore increased vaulting of the dorsum are part of the same process of compensatory changes in skull dimensions and brain shape described for the observed changes in the GB with craniosynostosis [9, 24] and head shape [27, 36] and will also result in changes to cervical conformation.

#### Protective conformation traits

The grid framework generated when the circle, lines and angles are combined together provides a visual and objective representation of the caudal fossa and craniocervical junction that pose less risk for SM. However the number and complexity of the measurements involved would be impractical and it is recommended that a specific imaging software program be developed which compares a user dog against standards known to reduce the risk of SM. Such a diagnostic tool might be used to create further models of CSF flow and model suitable surgical treatment. The morphometries would also provide information for genetic studies and estimated breeding values so that breeders can select the most suitable mates in their breeding programmes in order to reduce disease prevalence.

## Conclusion

This study used a range of morphometric measurements to characterize the hindbrain and craniocervical junction and successfully compared the phenotypic variables of Chihuahua, Affenpinscher and CKCS dogs with and without SM. It used refined morphometric techniques developed to investigate the genetic basis of CM in the GB. A total of 14 of the 23 variables considered were significant for SM and included the five traits found in the GB analysis, suggesting elements of a common aetiology. Different combinations and values of traits distinguished unique differences between the three study breeds. Two changes in angulation, the ‘sphenoid flexure’ rostral to the spheno-basioccipital synchondrosis and ‘cervical flexure’ which extended caudally to C2, were common to all three breeds. These flexures were associated with occipital bone hypoplasia and reduced caudal cranial fossa volume and, together or individually, they introduced risk factors to the severity for SM by compromising CSF flow dynamics and/or neural parenchyma compliance.

The complexity of quantifying the phenotype involved evokes the need to develop software in the form of a digital mapping tool that might be used to identify dogs at risk of SM and CM associated pain. Such a tool might assist with the diagnosis of different traits predisposing SM in order to consider alternative surgical management, for example, a ventral rather than dorsal decompression. Furthermore, the objective morphometries might provide estimated breeding values for screening breeding dogs and reduce the risk of SM through selective breeding.

## Supporting Information

S1 Morph Movie Chihuahua 1a with atlanto-occipital overlapping (AOO) but without SM that morphs to Chihuahua 1b with AOO and SM. The basioccipital and supraoccipital bones shorten. Angles 1 and 2 in the midbrain become smaller and the occipital crest reduces in size so that the cerebellum is deformed and invaginated under occipital lobes. The dens becomes more angled and moves closer to the foramen magnum resulting in a ventral flexure of the spinal cord. This conformational change is called ‘cervical flexure’. The obtuse angle 3 is associated with AOO.
(ZIP)

S2 Morph Movie Affenpinscher 2a without SM that morphs to Affenpinscher 2b with SM. The basicranium shortens and the midbrain angles 1 and 2 become smaller so that the cerebellum is deformed and flattened against the flattened and shortened supraoccipital bone and is invaginated under the occipital lobes. The occipital crest is also reduced. The dens appears to move closer towards the foramen magnum with greater angulation resulting in ‘cervical flexure’ of the spinal cord.
(ZIP)

S3 Morph Movie CKCS 3a without SM that morphs to CKCS 3b with SM. Both basioccipital and supraoccipital bones shorten. As the basicranium shortens, the midbrain angles1 and 2 become smaller so that the cerebellum is deformed and flattened against the straightened supraoccipital bone and is invaginated under the occipital lobes. The basisphenoid bone is flexed dorsocaudally (‘spenoid flexure). The dens and atlas move closer to the foramen magnum with a change in angulation of the spinal cord (cervical flexure) elevating the medulla oblongata (medullary kinking).
(ZIP)

S1 Table Canonical Discriminant Function Coefficients for 1) Breeds 2) SM used in the scatterplots (Fig 2). (XLSX)

S2 Table Canonical Discriminant Function Coefficients used in the scatter plots (Fig 3) *L* = angle. (ZIP)

S3 Table Morphometric values for the three study breeds. (XLSX)
